# What Is the Optimal Treatment for Liver Hydatid Cysts in Emergency and Elective Situations?

**DOI:** 10.5152/tjg.2024.23523

**Published:** 2024-02-01

**Authors:** Enver Zerem, Željko Puljiz, Boris Zdilar, Suad Kunosić, Admir Kurtcehajic, Omar Zerem

**Affiliations:** 1 Department of Medical Sciences, The Academy of Sciences and Arts of Bosnia and Herzegovina, Bistrik, Sarajevo, Bosnia and Herzegovina; 2 Department of Gastroenterology and Hepatology, University Clinical Center, Split, Croatia; 3 Department of Medicine, Croatian Military Academy, Ilica, Zagreb, Croatia; 4 Department of Physics, University of Tuzla Faculty of Natural Sciences and Mathematics, Tuzla, Bosnia and Herzegovina; 5 Department of Gastroenterology and Hepatology, Blue Medical Group, Tuzla, Bosnia and Herzegovina; 6 Department of Internal Medicine, Cantonal Hospital “Dr. Safet Mujić”, University of Mostar, Mostar, Bosnia and Herzegovina

Dear Editor,

Öter and Yalçın^1^ conducted a very interesting retrospective study to evaluate the efficacy and safety of surgical intervention and compare it with the percutaneous puncture, aspiration, injection, and re-aspiration (PAIR) method in the treatment of liver hydatid cyst (HC), with a special focus on pre-interventional and post-interventional complications. They concluded that “the classic open surgical operations, laparoscopic surgical operations, or PAIR methods, are safe and usable in the treatment of hydatid disease. It is necessary to choose the treatment according to the patient’s needs. The risk of recurrence after both methods should be kept in mind.”^1^

We generally agree with the conclusions of the study. However, since there are no randomized controlled trials directly comparing PAIR and surgical treatment of HC (according to the literature available to us), it is not possible to assess the superiority of one method versus the other based on scientific evidence. We agree with the authors that the choice of treatment should be adapted to each patient individually, but we consider that the applicability of these methods largely depends on the availability of specialized expertise and multidisciplinary teams dedicated to the treatment of hydatid disease in a given center.

We have quite a long and extensive experience in treating HC with and without complications using the ultrasound-guided percutaneous PAIR method (more than 500 patients over a period of longer than 20 years),^2-5^ and we find that this topic deserves some comments regarding the applicability of the surgical and PAIR methods in the treatment of HC.

In the Discussion section, the authors specified that “although interventional radiological methods can be applied for selected patients (especially Gharbi stages 1 and 2), surgery is the mainstay of treatment” and “stage 3-4 HC should be treated surgically.”^1^ However, in our experience, stage 3 and 4 HC (with or without complications) can also be successfully treated with the PAIR method, and this method should be considered as initial management in the treatment of most patients with HC located in the abdomen. The reason for this is that PAIR is a much simpler method compared to surgery (including laparoscopic surgical intervention) with lower trauma for patients and a method that can be performed without general anesthesia. Also, PAIR is practically possible in all patients regardless of HC location, including patients with multiple HC and pre-interventional and post-interventional complications (Figures 1 and 2). In addition, after the initial PAIR treatment, we can introduce a drainage catheter into the HC for the purpose of draining the contents created by the gradual disintegration of the HC or by dissolving the solid remains of the previously deviscerated HC (Figure 2). Besides, vigorous irrigation can be performed through the catheter, which helps to dissolve solid contents, as well as taking samples from the HC cavity for cytological and microbiological analysis and the instillation of medicaments. The fact is that immediately after the devisceration of HC by the PAIR method (especially in stages 3 and 4, according to Gharbi), drainage of necrotic tissue with a catheter is very poor. But, during treatment, the transition from solid necrotic tissue to more fluid content leads to a higher success rate of the evacuation of necrotic tissue and other pathological contents from the HC cavity. Therefore, we believe that surgery (including laparoscopic surgical intervention) may represent overtreatment, as the treatment of first choice in most patients with abdominal HC.

In conclusion, we would like to point out that, despite the fact that surgery is suitable and effective, we believe that the PAIR method has more advantages and should be considered as the first-choice option in the therapeutic spectrum for most patients with abdominal HC. Also, we consider that future studies should better determine the place of the PAIR method and surgery in the treatment of patients with HC.

## Figures and Tables

**Figure 1.**
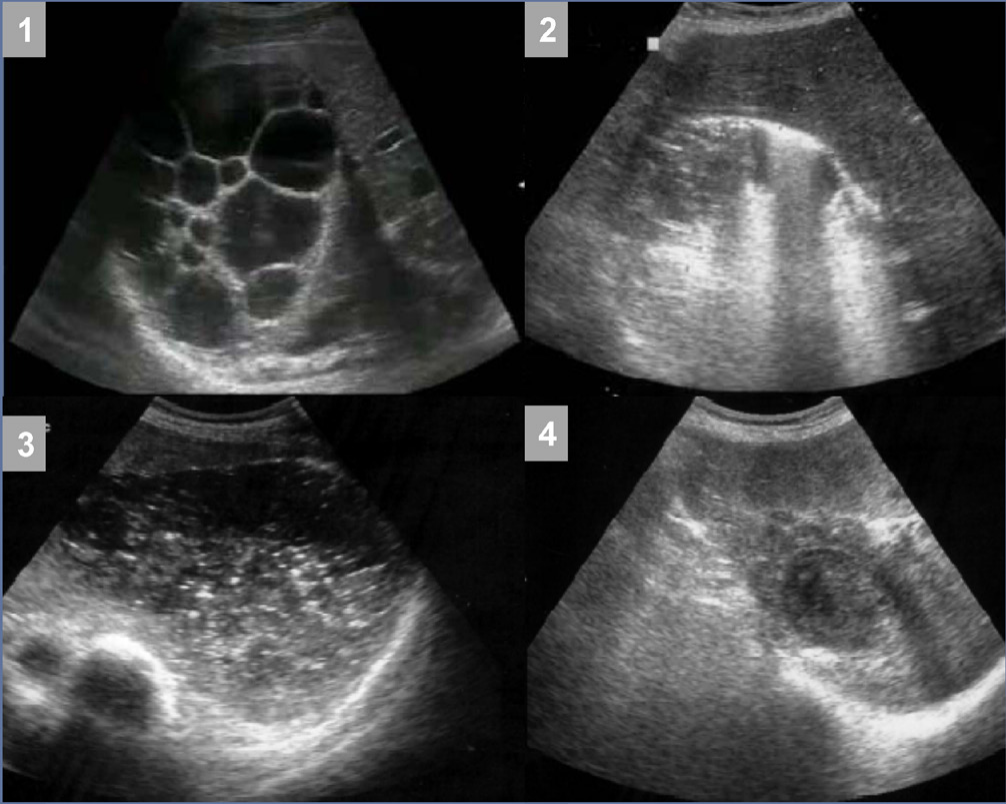
Devisceration of a large multivesicular hydatid liver cyst using the PAIR method with post-interventional complications. (1) Large multivesicular hydatid cyst in the right hepatic lobe. (2) The appearance of the same hydatid cyst immediately after instillation of the scolicidal agent. (3) An abscess in the cyst cavity that developed 5 weeks after PAIR and treated by percutaneous drainage. (4) A scar in the liver, at the site of the former hydatid cyst, 6 months after the procedure. PAIR, puncture, aspiration, injection, and re-aspiration.

**Figure 2.**
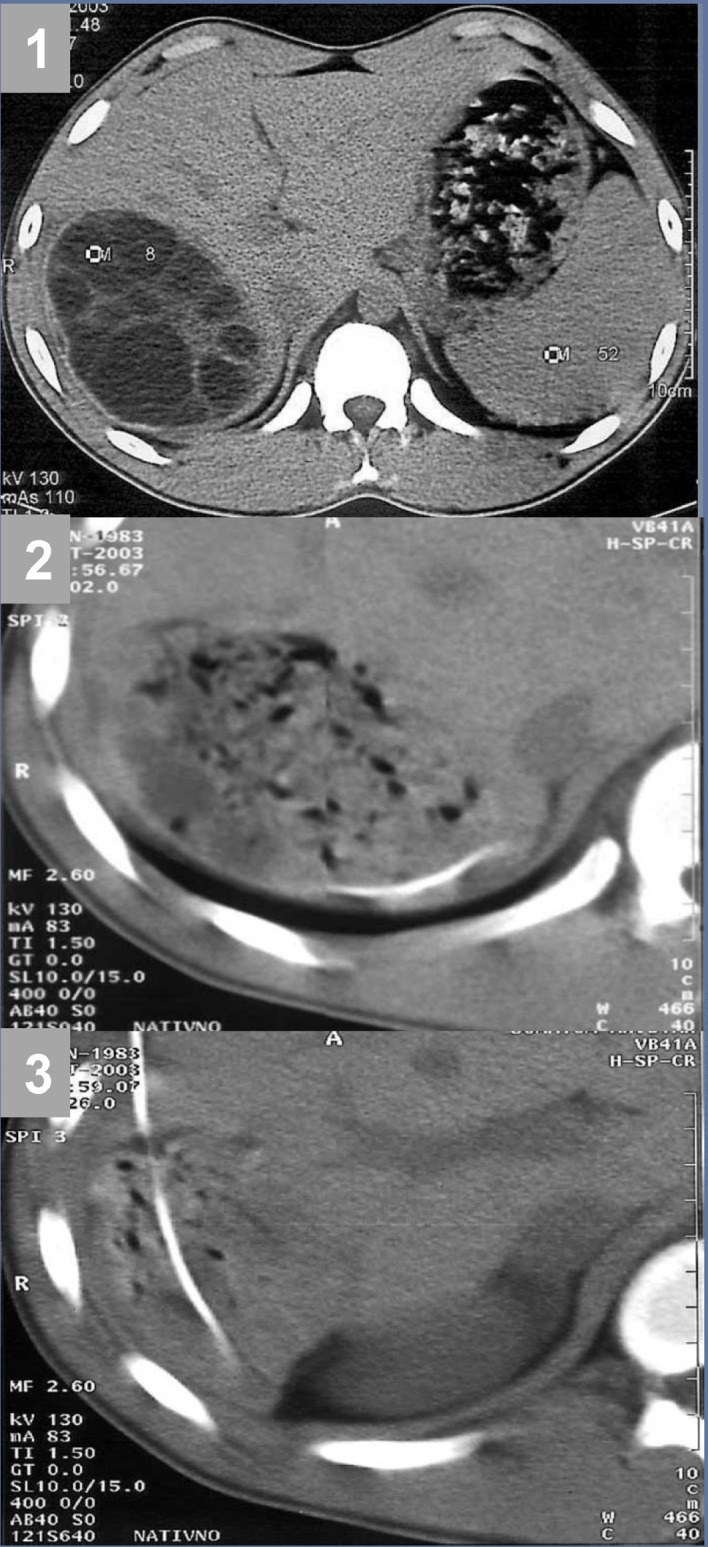
Drainage catheter in the hydatid cyst cavity after PAIR intervention. (1) Large multivesicular hydatid cyst in the right hepatic lobe. (2) The appearance of the hydatid cyst cavity after PAIR. (3) Drainage catheter in the hydatid cyst cavity. PAIR, puncture, aspiration, injection, and re-aspiration.
